# HIV-Tat upregulates the expression of senescence biomarkers in CD4^+^ T-cells

**DOI:** 10.3389/fimmu.2025.1568762

**Published:** 2025-04-24

**Authors:** Víctor Casanova, Andrea Rodríguez-Agustín, Rubén Ayala-Suárez, Elisa Moraga, María José Maleno, Josep Mallolas, Esteban Martínez, Sonsoles Sánchez-Palomino, José M. Miró, José Alcamí, Núria Climent

**Affiliations:** ^1^ AIDS and HIV Infection Group, Fundació de Recerca Clínic Barcelona-Institut d’Investigacions Biomèdiques August Pi i Sunyer (FRCB-IDIBAPS), Barcelona, Spain; ^2^ Department of Medicine, Universitat de Barcelona (UB), Barcelona, Spain; ^3^ Infectious Diseases Unit, Hospital Clínic de Barcelona, University of Barcelona, Barcelona, Spain; ^4^ Centro de Investigación Biomédica en Red sobre Enfermedades Infecciosas (CIBERINFEC), Instituto de Salud Carlos III (ISCIII), Madrid, Spain; ^5^ Reial Academia de Medicina de Catalunya (RAMC), Barcelona, Spain; ^6^ AIDS Immunopathology Unit, Centro Nacional de Microbiología, Instituto de Salud Carlos III (ISCIII), Madrid, Spain

**Keywords:** HIV-Tat, HIV, cellular senescence, SASP, aging, CD4^+^ T-Cell

## Abstract

**Introduction:**

Current antiretroviral therapy (ART) for HIV infection reduces plasma viral loads to undetectable levels and has increased the life expectancy of people with HIV (PWH). However, this increased lifespan is accompanied by signs of accelerated aging and a higher prevalence of age-related comorbidities. Tat (Trans-Activator of Transcription) is a key protein for viral replication and pathogenesis. Tat is encoded by 2 exons, with the full-length Tat ranging from 86 to 101 aa (Tat_101_). Introducing a stop codon in position 73 generates a 1 exon, synthetic 72aa Tat (Tat_72_). Intracellular, full-length Tat activates the NF-κB pro-inflammatory pathway and increases antiapoptotic signals and ROS generation. These effects may initiate a cellular senescence program, characterized by cell cycle arrest, altered cell metabolism, and increased senescence-associated secretory phenotype (SASP) mediator release However, the precise role of HIV-Tat in inducing a cellular senescence program in CD4^+^ T-cells is currently unknown.

**Methods:**

Jurkat Tet_off_ cell lines stably transfected with Tat_72_, Tat_101_, or an empty vector were used. Flow cytometry and RT-qPCR were used to address senescence biomarkers, and 105 mediators were assessed in cell supernatants with an antibody-based membrane array. Key results obtained in Jurkat-Tat cells were addressed in primary, resting CD4^+^ T-cells by transient electroporation of HIV-Tat-FLAG plasmid DNA.

**Results:**

In the Jurkat cell model, expression of Tat_101_ increased the levels of the senescence biomarkers BCL-2, CD87, and p21, and increased the release of sCD30, PDGF-AA, and sCD31, among other factors. Tat_101_ upregulated CD30 and CD31 co-expression in the Jurkat cell surface, distinguishing these cells from Tat_72_ and Tet_off_ Jurkats. The percentage of p21^+^, p16^+^, and γ-H2AX^+^ cells were higher in Tat-expressing CD4^+^ T-cells, detected as a FLAG^+^ population compared to their FLAG^-^ (Tat negative) counterparts. Increased levels of sCD31 and sCD26 were also detected in electroporated CD4^+^ T-cell supernatants.

**Discussion:**

Intracellular, full-length HIV-Tat expression increases several senescence biomarkers in Jurkat and CD4^+^ T-cells, and SASP/Aging mediators in cell supernatants. Intracellular HIV-Tat may initiate a cellular senescence program, contributing to the premature aging phenotype observed in PWH.

## Graphical Abstract

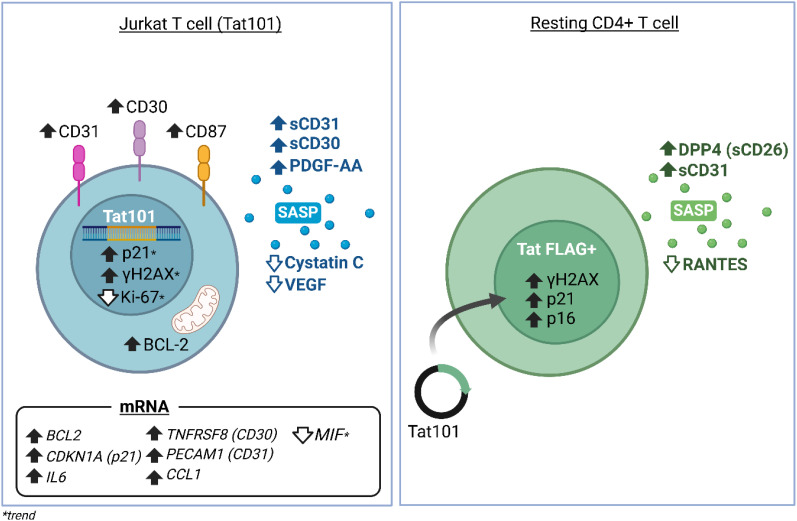

## 1 Introduction

Current antiretroviral therapy (ART) has transformed HIV from a progressive and fatal infection into a manageable chronic condition. ART has enabled people with HIV (PWH) to live longer, with estimations showing that more than 70% of PWH will be over 50 years of age by 2030 (1, 2). Despite ART success, PWH have a higher prevalence of age-associated comorbidities like cancer, cardiovascular diseases, frailty, and neurocognitive impairment than those without HIV (3, 4). Importantly, up to 64% of PWH of 50 years of age present 2 or more comorbidities, compared to 43% of HIV-negative, aged-matched controls (5). This phenotypic resemblance to the elderly led to the hypothesis that HIV infection may cause ‘premature aging’ (6, 7). Aging is defined as a functional decline in several organs and systems, leading to susceptibility to disease and death (8). The cumulative effects of HIV replication, chronic inflammation, and long exposures to ART treatment may contribute to the premature aging of PWH (9, 10).

Cellular senescence, one of the hallmarks of aging (8), is a complex cellular response to different stressors, such as oncogene activation (11, 12), irradiation, cytotoxic stimuli or oxidative stress, among others (13). Senescence was first defined in cultured lung fibroblasts that reached replicative exhaustion (14), where progressive telomere attrition is recognized by the DNA damage response (DDR) proteins γH2AX and 53BP1 (P53 pathway) (15), inducing the transcription of the cyclin-dependent kinase inhibitors *CDKN1A* (p21^CIP1^) and *CDKN2A* (p16^INK4A^) (16). This ultimately leads to cell cycle arrest and reduced expression of the proliferation marker *MKI67* (Ki-67) (16). Additionally, senescence involves profound morphological changes, chromatin reorganization, apoptotic resistance (BCL2), metabolic reprogramming (SA-β-Gal) and the establishment of a complex secretory program, termed the senescence-associated secretory phenotype (SASP) (17). The SASP includes cytokines, chemokines, angiogenic factors, proteases, collagens and other factors with a paracrine and inflammatory effect, able to induce senescence on surrounding cells (18–20). The exact composition and intensity of the SASP varies according to the senescent stimuli and cellular type. Cytokine induction leads to inflammation and the recruitment of immune cells that survey and clear senescent cells (21). This is beneficial in limiting tumorigenesis and promoting wound healing, however, aberrant accumulation of senescent cells occurs during aging and in many pathologies, contributing to chronic inflammation and organ damage (13).

In this regard, different hallmarks of cellular senescence have been detected in PBMCs from PWH, such as telomere shortening (22, 23), and increased p16^INK4A^ expression (24–26). BCL2, a senescence biomarker contributing to the apoptotic resistance seen in senescent cells, is also important for HIV infection, as it supports the survival of infected cells and HIV persistence (27). Preliminary results from our group (*Climent* et al, manuscript in preparation) indicate that cellular senescence biomarkers are upregulated in *ex-vivo* CD4^+^ T-cells from PWH during untreated HIV infection. Importantly, ART does not fully revert these changes after 1 year of treatment. HIV replication and viral products such as gp120, Nef or Tat have been proposed as potential inducers of cellular senescence in cells from HIV individuals (9). Only a few studies explore the role of HIV proteins in cellular senescence induction (reviewed in (28). Specifically, X4 and R5 HIV gp120 proteins increase SA-*β*-gal staining in endothelial cells (29, 30). Nef increases oxidative stress and mitochondrial dysfunction in human bone marrow mesenchymal stem cells (31) and SA-*β*-gal Levels, p16 and p53 activity in human adipose tissue (32), globally inducing early senescence in those cells. Finally, chronic exposure of human bone marrow mesenchymal stem cells to p55-gag results in reduced cell proliferation and increased senescence biomarker expression (33). Interestingly, most of these effects have also been observed in the presence of HIV-Tat, mostly due to increased NF-κB pathway signaling and ROS generation (29, 31, 32, 34, 35).

Tat is a regulatory protein expressed early in HIV transcription. It is encoded by two exons that translate into a 101-residue protein (Tat_101_ or full-length Tat). Tat is critical for viral replication by facilitating efficient elongation of viral transcripts through binding to the RNA polymerase II (RNAPII) complex and recruiting cellular elongation factors (36). HIV-Tat is also actively secreted by HIV‐1‐infected cells to the extracellular space where it mediates additional effects on surrounding cells (37). The first exon of Tat comprises amino acids from 1–72 (Tat_72_) and introducing a stop codon in position 73 produces an active protein (Tat_72_) that partially maintains the elongation ability of the full-length Tat (Tat_101_) (38). The second exon, consisting of amino acids from 73–101, enhances the protein’s transcriptional competence and adds several additional functions. Previous work from our group showed that the presence of the second exon of Tat was necessary to increase NF-κB pathway signaling, BCL2 expression, resistance to FASL-mediated apoptosis, ROS generation, and changes in cytoskeleton organization both in Jurkat T-cell models and primary CD4^+^ T-cells (38–42). Interestingly, some of these changes are now considered hallmarks of cellular senescence (43). Ultimately, full-length Tat has been shown to reprogram CD4^+^ T-cells by directly regulating transcription of over 400 genes (44).

In this regard, Tat has been shown to induce cellular senescence in endothelial cells (29), microglia (35), bone marrow mesenchymal stem cells (31) and human adipose tissue (32). Furthermore, Tat can be detected in the serum (45) and spinal fluid (46) of ART-treated, and virally suppressed PWH. Even low doses of a chronic Tat expression are sufficient to cause a neurodegenerative phenotype (47) and neuronal age-related diseases (48) in mouse models, underscoring the importance of Tat effects even in the absence of full viral replication. However, the specific impact of the different Tat forms in eliciting a CD4^+^ T-cell senescence program is currently unknown. Given the above, we wanted to explore the role of Tat in inducing the cellular senescence program in CD4^+^ T cells.

We hypothesized that full-length Tat expression in CD4^+^ T-cells would lead to the onset of cellular senescence. To this end, we addressed the effect of Tat_72_ or Tat_101_ on the expression of several canonical senescence biomarkers encompassing different biological characteristics of senescent cells, according to the SenNet recommendations (43). We addressed BCL2, p21^CIP1^, p16^INK4A^, Ki-67, γH2AX, CD87/uPAR, SA-βGAL, and an array of SASP factors both at the mRNA and at the protein level, in an established and well-characterized Jurkat cell model stably expressing Tat (38, 39, 41, 42). We validated these important changes in primary CD4^+^ T-cells. We show that full-length Tat increases critical senescence biomarkers and SASP factors. We propose that full-length Tat expression may contribute to chronic inflammation and the HIV premature aging phenotype seen in PWH.

## 2 Material and methods

### 2.1 Cell lines

Jurkat-Tat_72_ and Jurkat-Tat_101_ stably express HIV-Tat first exon (1 exon, 1-72aa; Tat_72_) or full-length HIV-Tat (2 exons, 1-101aa; Tat_101_), respectively. These stable transfectants were created and obtained from Alcamí and Coiras’ lab (Instituto de Salud Carlos III, Madrid, Spain). Shortly, stable transfectants were generated by electroporation of pTRE2hyg-Tat_72_, pTRE2hyg-Tat_101,_ or a pTRE2hyg empty vector (control; Tet_off_) in the Jurkat-TET_off_ cell line (Clontech, BD Biosciences) and stabilized with hygromycin B. In this TET_off_ system, Tat expression can be repressed by adding 1 μg/ml of Doxycycline (DOX) (Takara Bio, Mountain View, CA, USA) for 48 h. Tat expression in these cells and DOX silencing have been extensively characterized before (38, 39, 41, 42, 49). Jurkat cells were grown in RPMI 1640 medium supplemented with 10% (v/v) fetal calf serum (FCS), 2 mM l-glutamine, 100 μg/ml streptomycin, and 100 U/ml penicillin (GIBCO, Thermo Fisher, Waltham, MA USA), termed R10 media. Culture media in Jurkat-Tat cells was supplemented with 300 μg/ml Geneticin (G418 Sulfate) and 300 μg/ml Hygromycin B (GIBCO). Cells were maintained in a humidified air 5% CO_2_ atmosphere at 37°C.

### 2.2 Primary CD4^+^ T-cells

Peripheral blood mononuclear cells (PBMCs) from buffy coats (Banc de Sang i Teixits, Barcelona, Spain) were isolated by density-gradient centrifugation (Lymphoprep, Stem Cell Technologies, Vancouver, BC, Canada), at 800g for 30 min at room temperature. The collected PBMCs were then washed twice with phosphate-buffered saline (PBS, Corning, Glendale, AZ, USA), counted, and viability addressed with an automatic counter Luna FL system (Logos Biosystems, Villeneuve d’Ascq, France) using an Acridine Orange/Propidium Iodide dual stain. CD4^+^ T-cells were negatively isolated using the EasySep™ Human CD4^+^ T Cell Enrichment Kit (Stem Cell Technology), following the manufacturer’s instructions. The purity and viability of isolated CD4^+^ T-cells were routinely checked by flow cytometry and found to be above 93% and 90% respectively.

### 2.3 Plasmids

Long terminal repeat (LTR)-LUC and LTR-GFP plasmids were obtained from Alcami’s lab and were previously described (38, 39, 50). pEGFP-N1 plasmid was a gift from Alcami’s lab and was originally from Clontech (BD Biosciences). For transient transfections of HIV-Tat, the same Tat_72_ and Tat_101_ cDNA sequences used to generate the stable Jurkat-Tat transfectants, were cloned into pcDNA3.1(+) backbones using Genescript Express Cloning services (Genscript, Oxford, UK). pcDNA3.1(+) -Tat_72_, a pcDNA3.1(+) -Tat_101,_ and these two same constructs but with a C-Terminal DYKDDDDK (FLAG) tag fused to HIV-Tat were generated, yielding pcDNA3.1(+) -Tat_72-DYK_ and pcDNA3.1(+) -Tat_101-DYK_ plasmids. An identical, empty pcDNA3.1(+) vector was used as a control plasmid. Heat shock transformation of competent bacteria (Library Efficiency DH5α, Invitrogen, Waltham, MA, USA) was used to amplify all DNA plasmids, which were purified using a PureYield™ Plasmid Maxiprep System (Promega Corporation, WI, USA). Nucleic acid concentrations were determined based on 260nm absorbance with an EzDrop 1000 Spectrophotometer (Blue-Ray Biotech, New Taipei City, Taiwan).

### 2.4 Cell electroporation

Cells were electroporated using a NEON NXT device (Invitrogen). Jurkat cells were split and grown in fresh media the day before electroporation, then washed twice with PBS and seeded at 2×10^7^ cells/ml in R-Buffer, following manufacturer recommendations. For Neon 10 μl Tips, a total of 2×10^5^ cells and 0.75 μg of LTR-GFP DNA or pEGFP-N1 plasmid were used in 10 μl R-Buffer. Jurkat cells were electroporated using 1325V, 10ms and three pulses and immediately placed in 0.5 ml pre-warmed RPMI, 10% FCS antibiotic-free media in a 24-well plate culture vessel. 24 h post-electroporation, GFP expression and cell viability were addressed by flow cytometry. LTR-GFP was used to address Tat transactivation and the pEGFP-N1 plasmid was used as an indicator of transfection efficiency.

A total of 2×10^6^ CD4 cells and 7.5 μg of empty pcDNA3.1(+), pcDNA3.1(+) -Tat_72-DYK_, or pcDNA3.1(+) -Tat_101-DYK_ DNA plasmids were electroporated in T buffer with a 100 μl NEON NXT tip using 2200V 20MS in one pulse (51). Immediately after the transfection, CD4^+^ T-cells were placed in pre-warmed RPMI, 10% FCS, antibiotic-free culture media. Transfection efficiency and viability was measured with a pEGFP-N1 plasmid as indicated above.

### 2.5 RNA isolation, RT-qPCR

Jurkat and electroporated CD4^+^ T-cell pellets were harvested and immediately frozen at the indicated times. Total RNA was isolated using the RNAeasy mini kit (Qiagen, Hilden, Germany) and the genomic DNA removed with an on-column DNAse incubation step (RNase-Free DNase Set; Qiagen). RNA concentrations were determined using an EzDrop 1000 Spectrophotometer (Blue-Ray Biotech) and integrity checked with a TapeStation RNA ScreenTape (Agilent, Madrid, Spain). Total RNA (0.5 μg for Jurkat cells; 0.15 μg for electroporated CD4^+^ T-cells) was transcribed to cDNA with a SuperScript™ IV VILO RT mastermix (Invitrogen) in a 20 μl reaction, following the manufacturer’s instructions. 1 μl from cDNA synthesis reaction and 5 μl Fast Advanced Taqman MasterMix were used in a total 10μl FAST-qPCR reaction in 0.1 ml MicroAmp Fast Optical 96-well plate (Applied Biosystems, Foster City, US) and fluorescence signal detected with a StepOne Plus instrument (Applied Biosystems). Predesigned FAM-MGB Taqman primers and probes (Applied Biosystems) were used to detect senescence markers and are listed in **Table 1**. The following custom FAM-MGB TaqMan primers and probe were synthesized (ThermoFisher) and used to detect both forms of HIV-TAT mRNA:

**Table 1:** Predesigned FAM-MGB Taqman primers and probes used in this study.

Target Gene	Assay ID	Gene name/Aliases
***CDKN1A***	Hs99999142_m1	P21^CIP1/WAF1^
***SERPINE1***	Hs01126606_m1	PAI-1
***GAPDH***	Hs99999905_m1	GAPDH
***ACTB***	Hs03023943_g1	BETA ACTIN
***IL6***	Hs00174131_m1	IL-6, Interleukin 6
***PLAUR***	Hs00182181_m1	CD87, UPAR
***BCL2***	Hs00608023_m1	BCL-2
***CDKN2A***	Hs00923894_m1	P16^INK4A^
***TNFRSF8***	Hs00174277_m1	CD30
***PECAM1***	Hs01065279_m1	CD31
***MIF***	Hs00236988_g	MIF
***VEGFA***	Hs00900055_m1	VEGFA
***CCL1***	Hs00171072_m1	CCL1

Forward 5’-TAGAGCCCTGGAAGCATCCAGGAAG-3’

Reverse 5’-CTATGCTCTGATAGAGAAGCT-3’

Probe: 5’-TGGCAGGAAGAAGCGGAGA-3’

The 2^-ΔΔCt^ method was used to quantify relative mRNA changes against GAPDH. Data is represented as fold changes against TET_off_ control.

### 2.6 Droplet digital PCR

CD4^+^ T-cells were isolated from cryopreserved PBMCs from PWH with EasySep™ Human CD4+ T Cell Enrichment Kit (StemCell Technologies). Cell activation was performed with Dynabeads™ Human T-Expander CD3/CD28 (Gibco), using 10μl of beads per 1×10^6^ cells, and 100U/ml IL-2. Retrotranscription of RNA and cDNA amplification was performed with the One-Step RT-ddPCR Advanced Kit for Probes (Bio-Rad). To detect Tat mRNA, we used the well characterized Tat/Rev primers and probe (FAM) (52) with an annealing temperature of 54°C. To detect Tat mRNA in stable Jurkats we used our Tat72 Taqman primers and probe (FAM), modified from (38) and described above. Every well contained a control of cellular mRNA presence for CD3 gene as housekeeping (HEX). Analysis was performed by adjusting ddPCR RNA raw data concentrations with RNA quantity and concentration in each well. The ddPCR was performed in a QX600 Droplet Digital PCR System (Bio-Rad).

### 2.7 SASP in cell supernatants

The different Jurkat cell lines were seeded at 0.5×10^6^ cells/ml in a round bottom 96-well plate for 3 days. Plates were then centrifuged at 300 x *g*, 5 min and cell supernatant stored at -80°C for further use. Mediator levels of 105 human soluble cytokines were addressed in Jurkat supernatants using a Proteome Profiler Human XL Cytokine Array Kit (R&D Systems, Biotechne, Abingdon, UK), following the manufacturer’s instructions. The membranes were developed using SuperSignal™ West Atto reagents (Thermo) and immediately placed together in an Odyssey Fc instrument (LI-COR, Nebraska, USA), to generate a single image containing Tet_off_, Tat_72_ and Tat_101_ cytokine arrays captured together at different time intervals. Files created by Image Studio acquisition software were analyzed in Empiria Studio 3.0 software (LI-COR), with the built-in signal analysis tool. The signal of each pair of spots was calculated and corrected by subtracting the mean signal intensity from the defined background spots. Then, the mean signal intensity of each cytokine was normalized to the mean signal intensity of the 6 reference spots distributed in each corresponding membrane.

For absolute quantification of sCD31, and sCD30 levels in cell-supernatants, a CD31 (PECAM-1) Human ProcartaPlex™ Simplex Kit, and a CD30 Human ProcartaPlex™ Simplex Kit were used, following the manufacturer’s instructions (Thermo Fisher Scientific). The assay was measured using a Luminex™ 200 Instrument System (Thermo Fisher Scientific).

### 2.8 Flow cytometry

4×10^5^ Jurkat cells were grown in 1 ml R10 medium in 24-well plates for 24 h. Cells were collected in FACS tubes and washed 2 times with PBS before Live/Dead Near IR or V450 staining (Invitrogen). Live/Dead staining was included in all flow cytometry measurements. After washing with PBS and blocking Fc Receptors (Trustain X FC, Biolegend, San Diego, US), the expression of surface markers CD87, CD30 or CD31 was addressed with the indicated monoclonal antibodies (**Table 2**). To further assess the intracellular markers BCL2 and γH2AX, cells were fixed and permeabilized using the Cytofix/Cytoperm kit (BD biosciences) following the manufacturer’s instructions. To assess the levels of the proliferation marker Ki-67, after Live/Dead staining, cells were fixed with ice-cold 70% ethanol and placed at -20°C for a maximum of 1 week. Cells were then washed twice with 2 ml of PBS 0.1% bovine serum albumin (BSA, Sigma-Aldrich) and incubated with FxCycle™ Violet Stain (Invitrogen) in PBS 0.1% Triton X-100 (Sigma-Aldrich) for 15 min. Cells were immediately analyzed with a FACS CANTO II (BD Biosciences). To assess intracellular p21 levels in Jurkat cells, ice-cold 90% Methanol fixation was used to fix and permeabilize cells after live/dead staining. Cells were stored at -20° C up to a week, then washed twice in PBS 0.1% tween-20, blocked with 1% human AB Serum and stained with a primary, unlabeled rabbit anti-p21 antibody (Abcam). After washing twice with PBS 2% FCS, a secondary anti-Rabbit-Alexa Fluor 488 antibody (Invitrogen) was used. Cells were analyzed using a Gallios Instrument (Beckman Coulter, California, US).

**Table 2:** Antibodies used for flow cytometry.

Name	Clone	Manufacturer	Reference	Application
CD87 APC	VIM5	Invitrogen	17-3879-42	Flow
CD87 BV650	V MA013	BD OptiBuild™	743098	Flow
BCL2 A647	100	Biolegend	658706	Flow
BCL2 BV421	100	Biolegend	658709	Flow
P16^ink4A^ PE	G175-1239	BD Pharmingen	556561	Flow
γH2AX PE	CR55T33	Invitrogen	12-9865-42	Flow
γH2AX PerCP-eFluor™ 710	CR55T33	Invitrogen	46-9865-42	Flow
CD3 PB	SP34-2	BD Pharmingen	558124	Flow
P21^Waf1/Cip1^ A647	12D1	Cell Signaling	1678587S	Flow
P21^Waf1/Cip1^	EPR362	Abcam	ab109520	Flow/WB
CD279 (PD1) BV786	EH12.1	BD-Horizon	563789	Flow
CD30 APC	BY88	Biolegend	333910	Flow
DYKDDDDK Tag A488	L5	Invitrogen	MA1-142-A488	Flow
KI67-PE	B56	BD Pharmingen™	51-36525X	Flow
CD4 BV510	SK3	BD Horizon™	562970	Flow
LIVE/DEAD™ Fixable Near IR (780)	N/A	Invitrogen™	L34992	Flow
LIVE/DEAD™ Fixable Violet	N/A	Invitrogen™	L34964	Flow

Electroporated CD4^+^ T-cells were harvested at 48 or 72 h post electroporation and washed twice with PBS before Live/Dead Near IR staining. Cell membrane CD4, CD87, PD1 and intracellular BCL2, γH2AX, p21, p16, and FLAG (HIV-Tat) expression levels were determined using the corresponding antibodies (**Table 2**). The eBioscience Foxp3/Transcription Factor Staining Buffer Set (Invitrogen) was used to fix and permeabilize cells allowing the detection of intranuclear antigens. The acquisition was performed on an Aurora Spectral Flow Cytometry (Cytek Biosciences B.V., The Netherlands) with a 4 lasers configuration (16V-14B-10YG-8R).

### 2.9 SA-β-gal staining

SA-β-gal-positive cells were detected using SPiDER-βGal (DOJINDO, Kumamoto, Japan, SG03), according to the manufacturer’s instructions. Specifically, Jurkat cells were incubated with bafilomycin A1 for 1 h at 37 °C and 5% CO_2_. After washing with PBS 1X, cells were incubated with SPiDER-βGal for 30 min at 37 °C 5% CO_2._

### 2.10 Fluorescence microscopy

Jurkat E6.1 cells were treated 24h with 0,25 μM etoposide and washed with 1XPBS. After that, SA**-**βGal staining was performed as described above (2.8 section in methods). Cells were then laid on Superfrost ultra plus^®^ microscope slides for 30 min at RT, salts were cleaned with deionized water and dried. Slides were cover-slipped with a drop of mounting medium containing DAPI for visualization of cell nuclei (ProLong Gold, Thermo Fisher Scientific). Finally, cells were observed using a 400x magnification under Nikon Eclipse E600 fluorescence microscope for green, red and blue fluorescence. Images were analyzed in ImageJ software.

### 2.11 Statistical analysis

Graphs were plotted using the GraphPad Prism 10.4.1 software (GraphPad Software, Inc., San Diego, California, USA). Data was subjected to normality tests. For multiple comparisons, a Repeated Measures One Way ANOVA followed by Tukey’s multiple comparison test was used. For all the tests used, a two tailed *P* value <0.05 was considered statistically significant.

## 3 Results

### 3.1 Tat_101_ expression increases cellular senescence protein biomarkers in Jurkat cells

To address whether HIV-Tat expression resulted in increased expression of canonical senescence biomarkers, we used a well-characterized Jurkat Tet_off_ model, stably expressing full-length Tat (Tat_101_), or Tat’s first exon (Tat_72_) (38, 41, 42, 49). As previously described (38, 41, 53), Jurkat Tat cell lines expressed comparable levels of TAT mRNA (**Supplementary Figure S1**). Interestingly, these levels were around 25-fold higher than activated CD4^+^ T-cells from PWH (**Supplementary Figure S1**). Despite being transformed cells, our Jurkat transfectants readily respond to senescence inducers (54) such as etoposide (ETO), that induce DNA damage. This results in increased γH2AX expression (55).Treating our cells with 0.25 μM ETO for 24h increased γH2AX, BCL2, and CD87 expression (**Supplementary Figures S2A, B, C**). SA-β-Gal staining after ETO treatment was also visualized in fluorescence microscopy (**Supplementary Figure S3**). The doses of ETO here used did not alter cell viability (**Supplementary Figure S4**).

**Figure 1:**
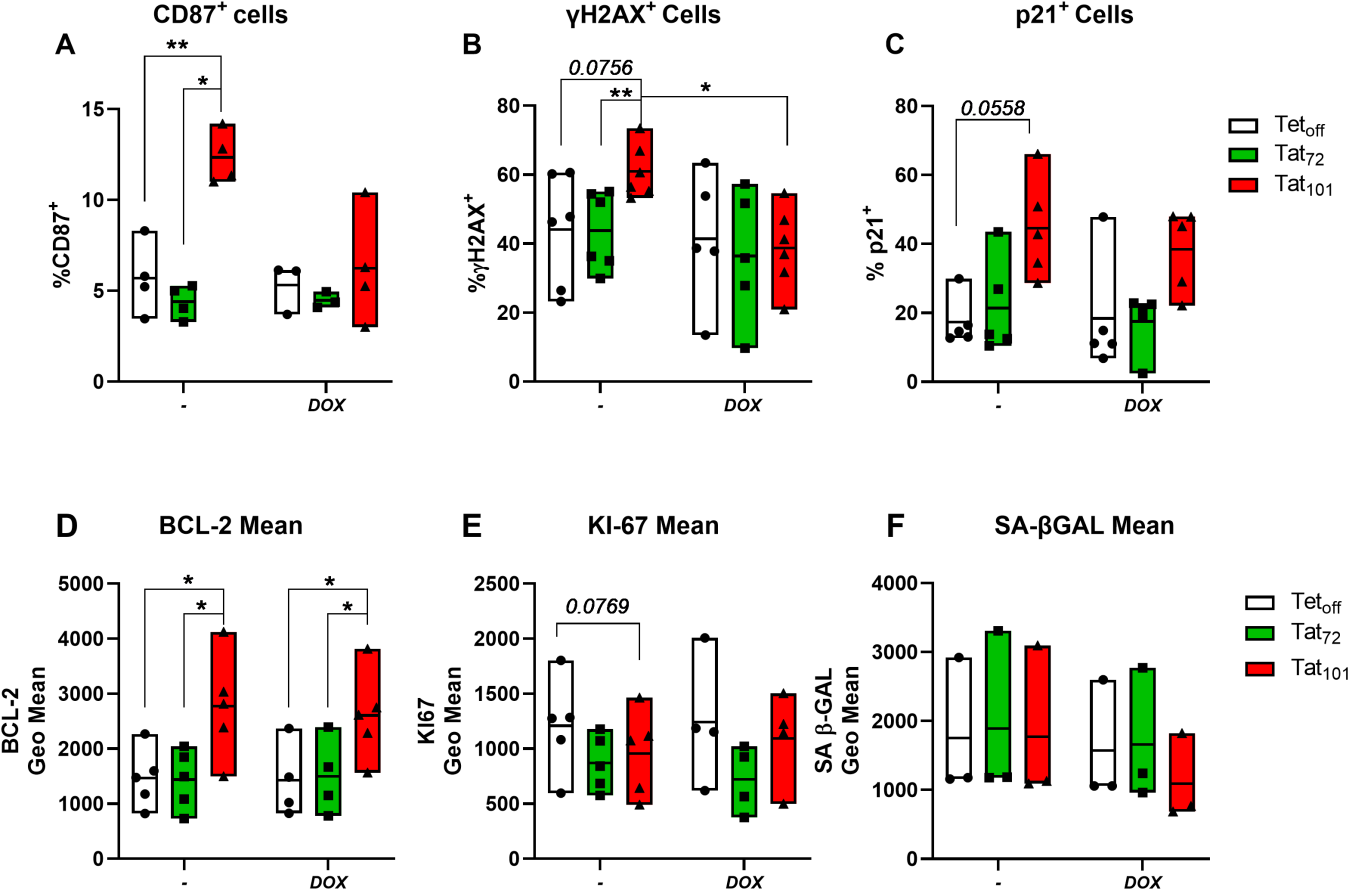
Full-length HIV-Tat increases the expression of senescence protein markers. Jurkat Tet_off_ T-cells stably expressing full-length HIV-Tat (Tat_101_) or first-exon Tat (Tat_72_) were treated with 1μg/ml Doxycycline for 48h (+DOX) or left untreated **(-)** and then the expression of senescence protein markers determined by flow cytometry. The percentage of live cells positive for CD87 **(A)**, γH2AX **(B)**, and p21 **(C)** and the geometric mean BCL-2 **(D)**, KI67 **(E)**, and Senescence associated β-GAL (SA β-GAL) **(F)** are shown. Symbols represent individual experiments (n=3-5) and floating bars indicate minimum to maximum values with a line at the mean value. *p ≤ 0.05, **p ≤ 0.01, ***p ≤ 0.001. Statistical analysis was performed by one-way ANOVA with Tukey’s multiple comparisons test.

Tat_101_ expression resulted in a statistically significant increase in the percentage of cells expressing CD87 compared to Tet_off_ controls and to Tat_72_-expressing cells (**Figure 1A**). Similarly, in all experiments the percentage of γH2AX^+^ and p21^+^ cells was higher in in Jurkat Tat_101_ than in Tet_off_ (p=0.0756; **Figure 1B** and p=0.0558; **Figure 1C**, respectively). Tat_101_ expression also increased BCL-2 geometric mean, compared to Tet_off_ and to Tat_72_ cells (**Figure 1D**). All experiments showed a trend towards a reduced expression of Ki-67 in Tat_101_ cells, compared to Tet_off_ (p=0.0769) (**Figure 1E** **).** Neither Tat_72_ nor Tat_101_ expression altered senescence-associated β-GAL activity (SA-β-Gal) (**Figure 1F**). There were no differences between Tat_72_ and Tet_off_ in any of these markers. Furthermore, DOX addition abrogated the Tat_101_-mediated increase in γH2AX^+^ cells (**Figure 1B**) and slightly reduced the percentage of CD87^+^ cells (p=0.0842) observed in Tat_101-_expressing cells (**Figure 1A**). Interestingly, DOX did not alter Tat_101_-mediated changes in BCL2 and only slightly reduced p21 levels. DOX addition did not alter cell viability at the times tested (**Supplementary Figure S3** **).**

### 3.2 Full-length, intracellular HIV-Tat increases levels of senescence-associated secretory phenotype mediators

**Figure 2:**
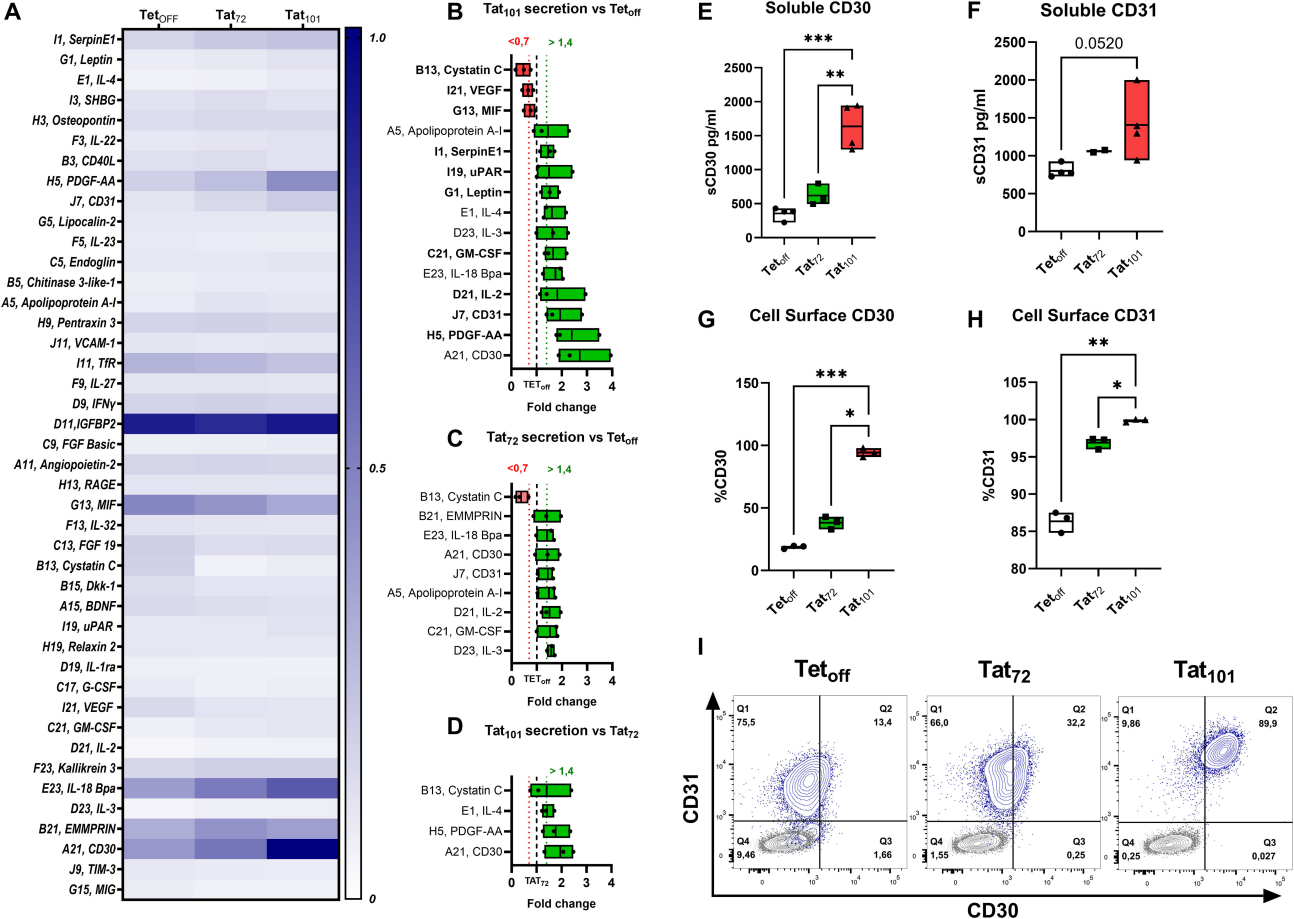
SASP Mediators in Jurkat cell line supernatants. Jurkat Tet_off,_ Tat_72_ or Tat_101,_ cells were grown in round bottom 96-well plates at 5×10^5^ cells/ml in 200 μl R10, and cell supernatants were collected after 72 hours. The expression levels of 105 cytokines were addressed with an antibody-based membrane array. **(A)** Heatmap indicates the mean intensity values of the 43 mediators with visible spots in the membrane after ELC incubation. Mediators with mean signals over 1.4 (green boxes) or below 0.7-fold change (red boxes) expression in Tat_101_ over Tet_off_ (dotted line) are shown in **(B)**. Changes in Tat_72_ over Tet_off_ **(C)** and Tat_101_ over Tat_72_ **(D)** are also shown. (n=3). Secreted levels of CD30 **(E)** and CD31 **(F)** were evaluated in Tet_off_ (n=4), Tat_72_ (n=2-3) and Tat_101_ by Luminex on the same cell supernatants as **(A)**. The percentage of cells expressing cell surface CD30 **(G)** or CD31 **(H)** was addressed by Flow Cytometry. Graphs indicate percentages of cells within the Live cell gate after 48h cell culture. CD30 and CD31 co-expression for each of the cell lines (blue contour plot) overlayed with autofluorescence controls (grey contour plot) from a representative experiment is shown in **(I)**. Symbols represent individual experiments (n=3), and floating bars indicate minimum to maximum values with a line at the mean. Statistical analysis was performed by one-way ANOVA with Tukey’s multiple comparisons test *p ≤ 0.05, **p ≤ 0.01, ***p ≤ 0.001.

We analyzed the composition of the SASP using a membrane-based antibody array detecting 105 cytokines. Out of them, 43 were visually detectable and their mean relative levels for each cell line were calculated (**Figure 2A**, **Supplementary Figure S5**). 12 factors were upregulated at least 1.4-fold and 3 downregulated at least 0.7-fold in Tat_101_ cell supernatants compared to control Tet_off_ (**Figure 2B** **).** We found that CD30, PDGF-AA, and CD31 showed the highest increase over Tet_off,_ with a mean fold-change of 2.71, 2.4, and 1.93, respectively. Cystatin C and VEGF were the targets that showed the highest downregulation in Tat_101_ compared to Tet_off,_ displaying a mean fold change of 0.47 and 0.65 respectively (**Figure 2B** **).** Interestingly, 7 out of the 8 mediators upregulated at least 1.4-fold in Tat_72_ over Tet_off_ (**Figure 2C**) were the same as those seen in the Tat_101_ vs Tet_off_ comparison, but displaying a more modest increase. In Tat_72_, IL-3, GM-CSF and IL-2 showed the highest up-regulation with 1.57, 1.53 and 1.5-fold-change over Tet_off_, respectively. Only Cystatin C showed a greater downregulation in Tat_72_ over control cells (0.38-fold-change) compared to full-length Tat (**Figure 2C**). Accordingly, CD30, PDGF-AA, IL-4 and Cystatin C were the only mediators that increased over 1.4-fold in Tat_101_ compared to Tat_72_ (**Figure 2D** **).** Importantly, 10 out of the 15 factors changed in Tat_101_ over Tet_off_ have well-defined roles in cellular senescence or aging (highlighted in bold in **Figure 2C** **).** Next, we validated and quantified the increase in soluble CD30 (sCD30) and CD31 (sCD31) levels in Tat_101_ and in Tat_72_ compared to Tet_off_ cells using a highly sensitive Luminex technique. Levels of sCD30 increased from 355.7 pg/ml (as a mean) in Tet_off_ cells to 619.8 in Tat_72_ and to 1636 pg/ml in Tat_101_ (**Figure 2E**). Regarding sCD31, levels increased from 800.7 pg/ml (as a mean) in Tet_off_ cell lines, to 1061 pg/ml in Tat_72_ and to 1408 pg/ml in Tat_101_ expressing cells (**Figure 2F** **).** We also addressed the expression of CD30 and CD31 in the cell membrane of Jurkat Tat_101_. Flow cytometry data showed a stark increase in the percentage of CD30^+^ cells (**Figure 2G**) and CD31^+^ cells (**Figure 2H** **),** from 18.78% and 86.6% of Tet_off_ cells to 94.2% and 99.86% in Tat_101_ cells, respectively. The geometric mean of these markers also increased, with a 5,02-fold increase in CD30 (**Supplementary S5B**) and a 6,8-fold increase in CD31 (**Supplementary S5C**) in Tat_101_ cells compared to Tet_off_. Tat_72_ cells showed a modest increase over Tet_off_ in the percentage of cells expressing these markers (**Figure 2F, H** **).** Furthermore, combining both markers enables a clear identification of Tat_101_ cells as a separate population (**Figure 2I**). In this regard, 89,9% of Tat_101_ cells stain positive for both CD30 and CD31 whereas only 13,4% and 32,2% are double positive in Tet_off_ and Tat_72_, respectively.

### 3.3 Tat_101_ increases the expression of senescence biomarkers at the mRNA level

**Figure 3:**
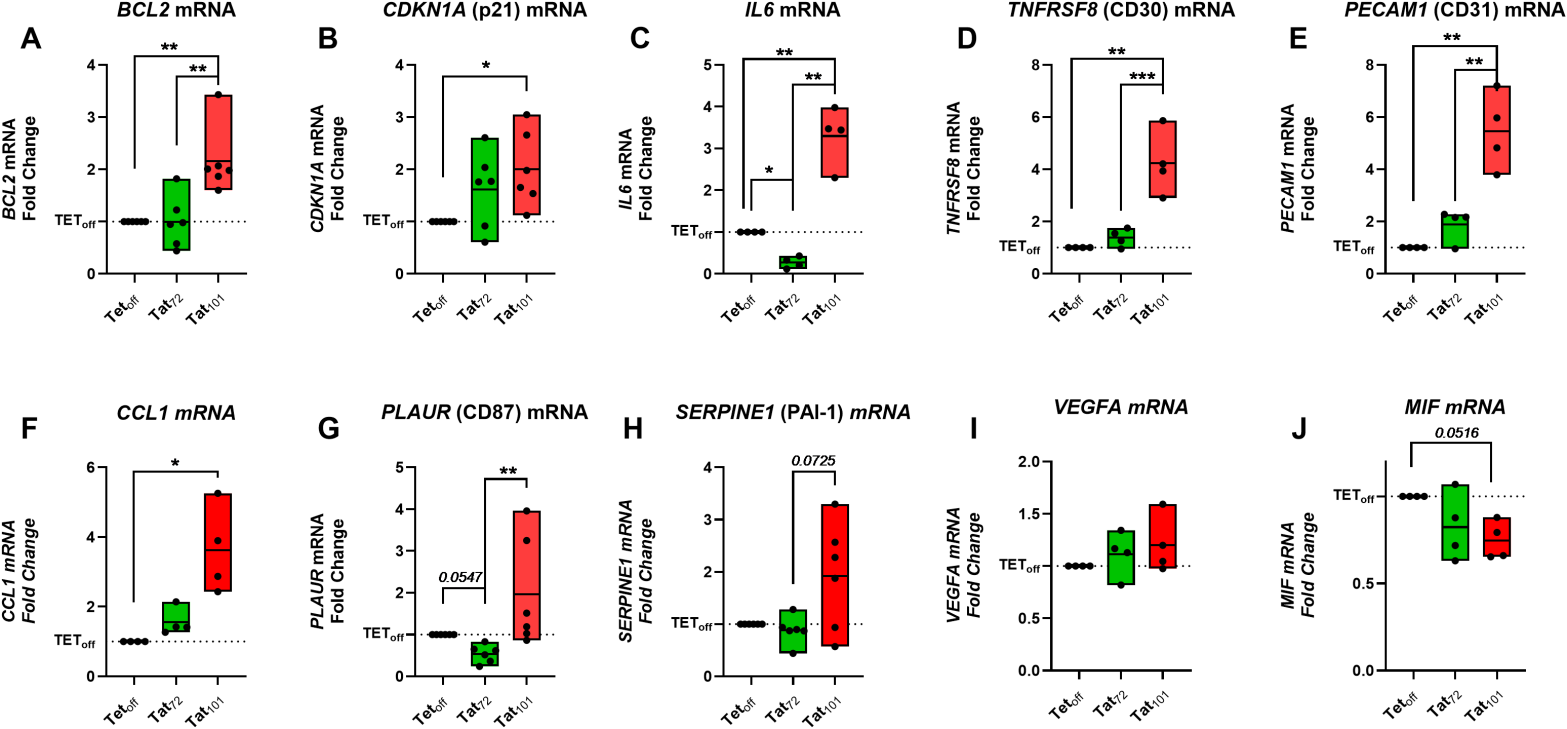
Full-length HIV-Tat increases several senescence markers at the mRNA level. Jurkat Tet_off,_ Tat_72_ or Tat_101,_ cells were grown for 24h in 24-well plates at 4×10^5^ cells/ml in 500 μl of R10. Cell pellets were collected, and mRNA levels of indicated senescent markers were determined by RT-qPCR **(A-J)**. Symbols represent individual experiments (n=4-5) with values representing the Fold Change versus Jurkat TET_off_ control cell line (dotted line) following the 2^-ΔΔCT^ calculation. Floating Bars indicate minimum to maximum values with a line at the mean value. *p ≤ 0.05, **p ≤ 0.01, ***p ≤ 0.001. Statistical analysis was performed by one-way ANOVA with Tukey’s multiple comparisons test on ΔCT values.

We aimed to evaluate the expression of the previously addressed senescence biomarkers and additional SASP factors at the mRNA level, using a Taqman RT-qPCR approach. We found that *BCL2, CDKN1A* (p21), *IL6, TNFRSF8* (CD30), *PECAM1* (CD31), and *CCL1* mRNA expression was significantly increased in Tat_101_ compared to Tet_off_ cell lines (**Figures 3A–F**). We noticed modest increases in *PLAUR* (CD87), *SERPINE* (PAI-1) and in *VEGFA* mRNA levels in Tat_101_ cell lines compared to Tet_off_, without statistical significance (**Figures 3G–I**) due to higher intragroup dispersion. A trend (p=0.0516) towards a reduced *MIF* mRNA expression was observed in Tat_101_ compared to Tet_off_. Tat_72_ expression did not significantly increase the senescence biomarkers here addressed when compared to Tet_off_ controls. The importance of the second exon is underscored by the fact that Tat_101_ expression significantly increased mRNA levels of *BCL2, IL6*, *TNFRSF8* (CD30), *PECAM1* (CD31) and *PLAUR (*CD87) mRNA over cells expressing Tat_72_. (**Figures 3A, B, D, E**). This data suggests that the second exon is important in inducing cellular senescence biomarkers at the transcriptional level.

**Figure 4:**
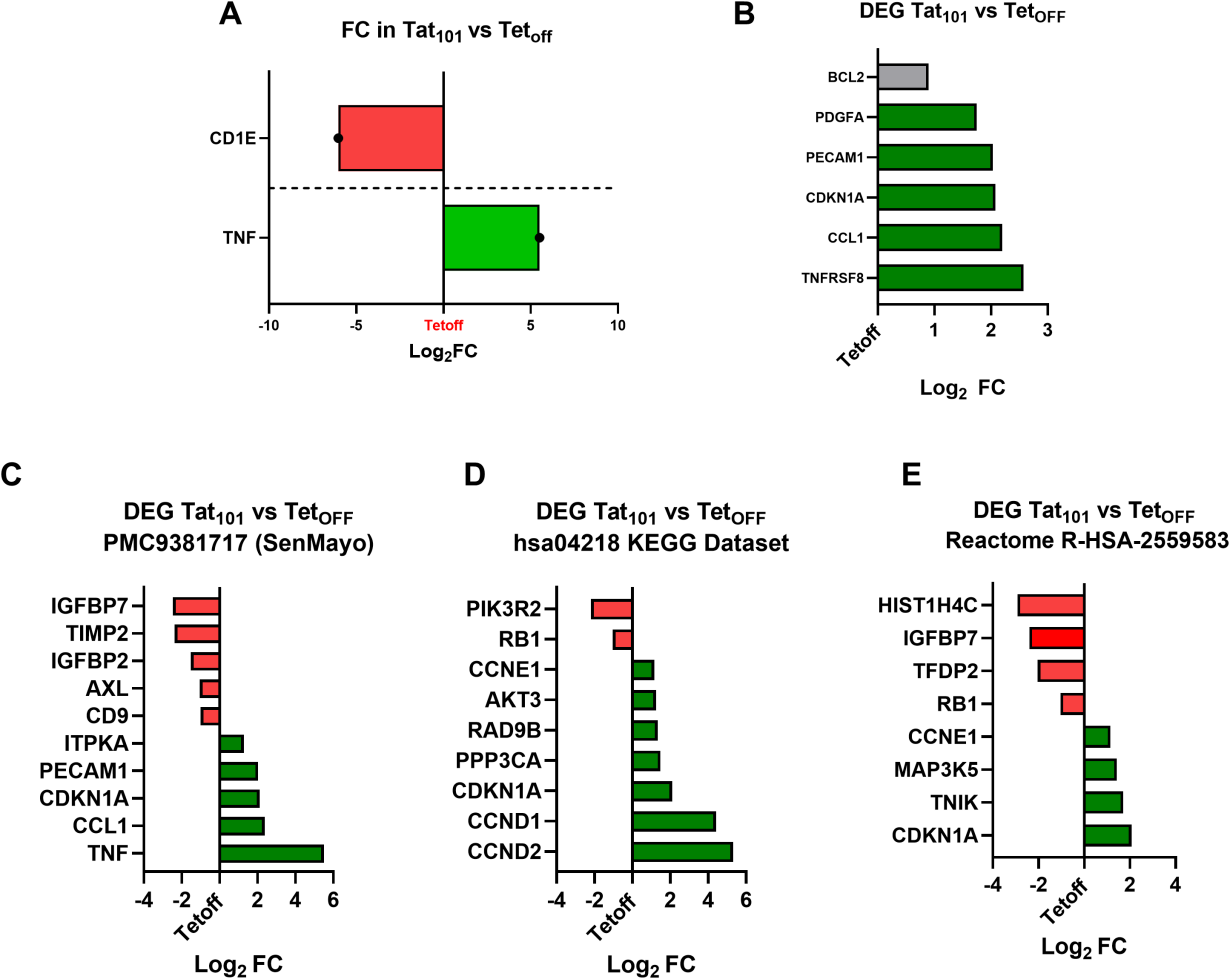
Full-length HIV-Tat increases transcripts involved in cellular senescence. An RNA-seq approach was used in the same Jurkat Tet_off,_ Tat_72_ or Tat_101,_ cells used here (*Rodríguez-Agustín*, under revision). Bars indicate the Log_2_ Fold Change **(FC)** in relevant DEG (Differentially Expressed Genes) in the Tat_101_ vs Tet_off_ comparison. Green bars indicate upregulated DEG Genes (**PPDE>0.95 and FC > 2)**, and red bars indicate downregulated DEG genes (PPDE>0.95, FC <0,5). **(A)** Tat_101_ stable expression increases *TNF* and decreases *CD1E* mRNA transcripts, well-known Tat target genes. **(B)** Senescence biomarkers and SASP targets addressed in this work that are also DEG in the RNA-seq experiment are shown. A grey bar indicates a DEG gene with an up-regulation below the 2-fold threshold (1, 8). Expression values (Log_2_FC) of Tat_101_ DEG genes present in the SenMayo dataset **(C)**, in the hsa04218 KEGG Dataset **(D)** or in the Reactome R-HSA-2559583 dataset **(E)** are shown.

To confirm and extend RNA results, we analyzed the expression of senescence associated-genes with RNA-Seq data generated in our companion paper (56). First, we tested well-known up-regulated (*TNF*) and down-regulated (*CD1E*) genes upon Tat expression (39, 44) (**Figure 4A** **).** *TNF* was upregulated 40-fold while *CD1E* was downregulated over 65-fold in Tat_101_ compared to Tet_off_, confirming known Tat-derived effects on these cell lines. We also found that *CCL1, CDKN1A, PECAM1, PDGFA*, and *TNFRSF8* were differentially expressed genes (DEG) in Tat_101_ compared to Tet_off_ (**Figure 4B**), supporting our previous mRNA and protein data. Next, DEG in the Tat_101_ vs Tet_off_ comparison, were compared to different senescence datasets available in the literature. We found that among the genes that comprise the SenMayo dataset (57), Tat_101_ expression upregulated *TNF, CCL1, CDKN1A, PECAM1*, and *ITPKA* mRNA levels while downregulated *IFGBPT7, IGFBP2, TIMP2, AXL*, and *CD9* (**Figure 4C** **).** We further compared DEG to other senescence datasets like hsa04218 (**Figure 4D**) and R-HSA-2559583 (**Figure 4E**), showing increased levels of senescence hallmarks such as *CCND2*, *CCND1, CCNE1*, or *AKT3* or decreased mRNA levels of *PIK3R2* and *RB1* in Tat_101_ cells. This information will prove valuable to understanding how full-length Tat alters cellular senescence pathways.

### 3.4 p21, p16 and γH2AX expression is increased in Tat-expressing (FLAG^+^) primary CD4^+^ T-cells

To address the role of HIV-Tat in cellular senescence in a non-transformed model, we used purified CD4^+^ T-cells, which are the primary target of HIV infection. Cells were electroporated with DNA plasmids (51) encoding full-length Tat or Tat’s first exon, fused to a C-terminal FLAG tag (DYK). Electroporation efficiency was around 16% at 24 hours post-electroporation (**Supplementary Figure S6A**) with cell viability at around 60% at this same time point (**Supplementary Figure S6B** **).** Nearly 3% of viable cells expressed Tat-_DYK_ proteins at 24 hours post-electroporation, as measured by FLAG detection (**Supplementary Figure S6C** **).** This was reduced to a mean of 0.5% at 72 h post electroporation in Tat_72-DYK_ (± 0.29%) and to around a mean of 1% in Tat_101-DYK_ (± 0.7503%) (**Supplementary Figure S6D**). Cell viability was similar between Tat_72-DYK_ (mean of 32.3% ± 9.1) and Tat_101-DYK_ (mean of 33.73% ± 14.42) (**Supplementary Figure S6E** **).** Additionally, we verified Tat expression with a Taqman qPCR approach, confirming a high expression of Tat mRNA at 24 hours, with a lower but still abundant expression at 72 hours (data not shown).

**Figure 5:**
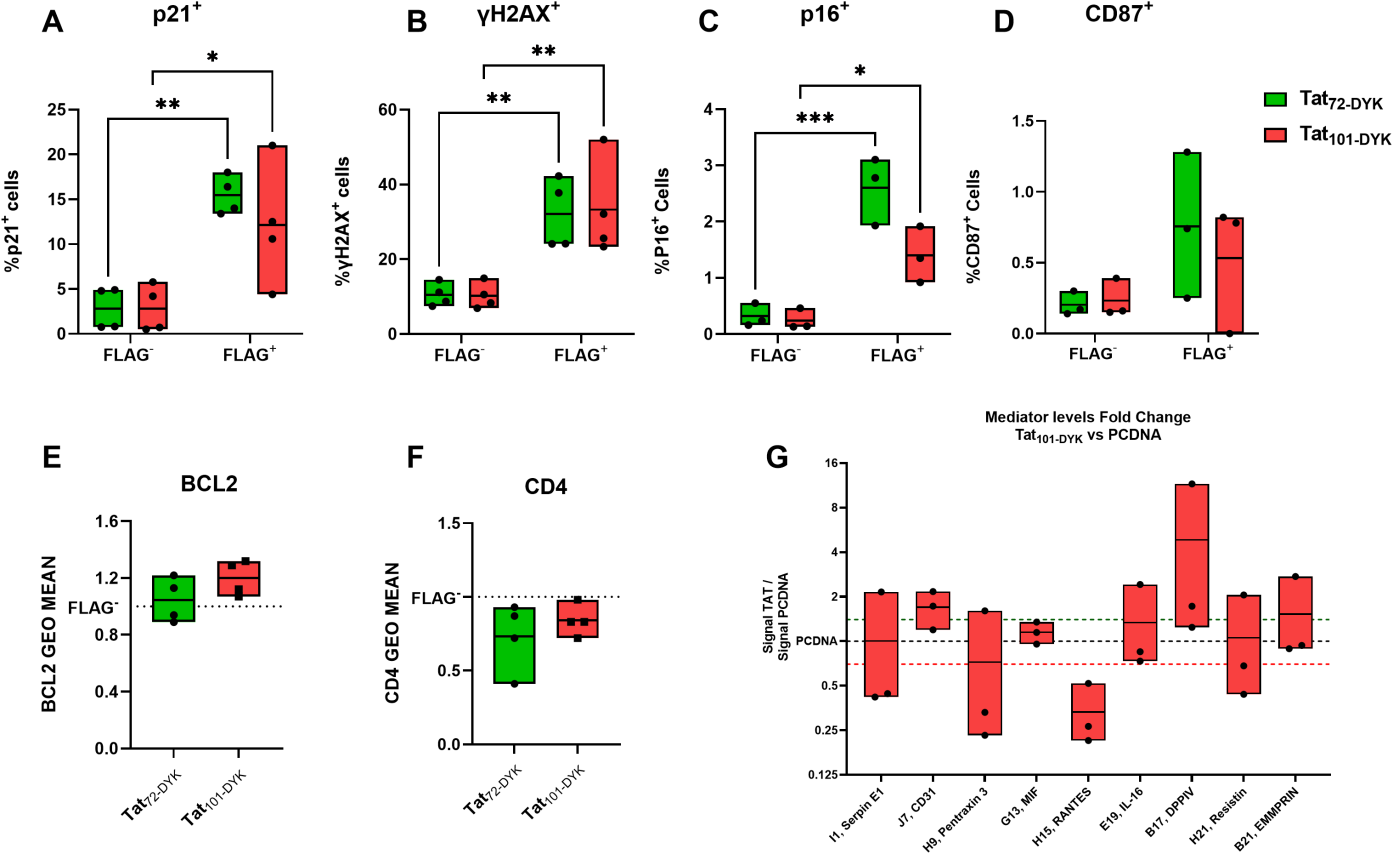
Tat-expressing CD4^+^ T-cells show increased levels of p21, γH2AX, and p16 senescence biomarkers. Resting CD4^+^T-cells isolated from buffy coats were transiently transfected with DNA plasmids encoding for Tat_72-DYK_, Tat_101-DYK_ or a pcDNA empty vector backbone. An anti-FLAG antibody was used to detect Tat-expressing cells. The percentage of p21^+^ **(A)**, γH2AX^+^ **(B)**, p16^+^ **(C)** and CD87^+^ **(D)** cells within viable (Live Dead Negative) FLAG^+^ or FLAG^-^ populations at 72hours post electroporation are shown. Fold change values in the geometric mean of BCL2 **(E)**, and CD4 **(F)** in FLAG^+^ cells compared to FLAG^-^ cells are indicated. An antibody membrane-based array was used to address changes in released mediators in cell supernatants. The levels (Fold-change) of detectable mediators in TAT_101-DYK_ cell supernatants against pcDNA cell supernatants are shown in **(G)**. Symbols represent individual experiments (n=3-4) and Floating Bars indicate minimum to maximum values with a line at the mean. A green dotted line indicates > 1.4-fold-change and a red dotted line indicates < 0.7-fold-change. *p ≤ 0.05, **p ≤ 0.01, ***p ≤ 0.001. Statistical analysis performed by two-way ANOVA with Tukey’s multiple comparisons test.

Expressing either form of Tat in CD4^+^ T-cells resulted in a marked increase in the percentage of p21^+^, γH2AX^+^, and p16^+^ cells in the FLAG^+^, (Tat-expressing cells) compared to their FLAG^-^ counterparts (**Figures 5A–C**). In addition, a similar trend was observed for the percentage of CD87^+^ cells (**Figure 5D**) but not reaching statistical significance. We also detected a marginal increase in BCL2 (**Figure 5E** **),** and a decrease in CD4 expression (**Figure 5F**) in FLAG^+^ cells compared to their FLAG^-^ counterparts, but these changes did not reach statistical significance (p=0.1796 and p= 0.2104, respectively). Taken together, this data confirms that Tat upregulates important senescence biomarkers in primary CD4^+^ T-cells.

### 3.5 HIV-Tat_101_ expression in resting CD4^+^ T-cells increases soluble CD31 and CD26 secretion in cell supernatants

We next aimed to determine whether Tat expression also resulted in increased SASP mediator secretion. Only 18 out of 105 mediators were visually detectable. Importantly, increased levels of CD31 and DPP4 (CD26) were detected in all experiments performed (**Figure 5G** **),** with 1.69 and 4.8-fold-change increases over pCDNA, respectively. In addition, we observed a 0.333-fold change (3.03-fold reduction) in the levels of RANTES (**Figure 5G** **).**

## 4 Discussion

Recent studies show that a broad range of viral infections, including lentiviral infections, result in the onset of a canonical cellular senescence program (58), reminiscent of other cellular responses to stress, such as oncogene-induced senescence (11). Growing pieces of evidence suggest that HIV infection induces cellular senescence, as an *in vitro* HIV infection of primary human fetal microglia results in increased SA‐β‐gal and p21 expression and IL-6 and IL-8 secretion (59). In addition, *in vitro* HIV infection activates the DNA damage response (DDR), increasing γH2AX and 53BP1 staining in U2OS cells (60) and CD4^+^ T-cells (61). In *ex vivo* T-cells from PWH, an elevated percentage of p16^INK4A^ -positive cells and reduced telomere length are observed in untreated PWH (19, 25, 26). We show here that full-length, intracellular Tat expression upregulates p21^CIP^, p16^INK4A^, CD87, γH2AX, and BCL-2 expression, which translates to increased expression of key cell cycle inhibitors and enhanced resistance to apoptosis. Additionally, the secretion of several SASP mediators is increased, shaping a pro-inflammatory secretome. These changes in Tat-expressing cells are characteristics of cellular senescence (13, 43). This supports the important role of Tat in the increased senescence markers observed in HIV infection and in PWH.

Our experimental model consists of a well-characterized Jurkat Tet_off_ cell line, stably expressing 1 or 2 exons forms of Tat (38, 39, 42, 49, 53). The 2 exons, 101aa form of Tat is the most common in clinical HIV isolates, while some strains encode for a 2 exon, 86aa Tat form (62). Exogenous delivery of an 86aa (32, 35) or a 101aa (31) form of soluble, recombinant HIV-Tat resulted in reduced cellular proliferation, increased SA‐β‐gal and p21 expression together with augmented IL-6 and IL-8 SASP secretion in cell cultures of human microglia (35), adipose tissue (32) and bone marrow mesenchymal stem cells (31). In our model, Tat is produced intracellularly, but we cannot exclude the possibility of Tat being released to the extracellular space. In our Jurkat model, we observe increased p21 protein and mRNA levels and elevated IL-6 mRNA production. In addition, we detect increased γH2AX in Tat_101_-expressing cells, which suggests a cellular response to DNA damage. Exogenous, recombinant HIV-Tat has been shown to increase ROS levels and increased DDR in primary B-cells, as detected by increased γH2AX levels (63). A similar finding is reported in an immortalized lymphoblastoid B cell line RPMI-8866 (64). Previous results showed increased ROS generation upon Tat_101_ expression in the same Jurkat cell model used here (41), which indicates an active DDR response. Importantly, the DDR response is known to elevate the transcriptional activity of *CDKN1A* (p21^CIP1^) and *CDKN2A* (p16^INK4A^) genes (15). We have also addressed the levels of CD87/uPAR, a novel, cell-surface biomarker of senescence (65), also secreted by senescent cells. Soluble uPAR/CD87 (suPAR) was among the first SASP factors identified (18). *In vitro* HIV infection leads to increased membrane and secreted CD87 expression (66, 67), and elevated levels of serum CD87 in PWH correlate with poor survival (68). Thus, increases in CD87 during HIV infection may be linked to Tat activity.

A critical feature of cellular senescence is SASP. Here, we report that Tat_101_ increases the secretion of known SASP mediators like PAI-1 (SerpinE1), suPAR, Leptin, GM-CSF and IL-2 (18, 69). In addition, CD30 and IL-18Bpa are considered markers of healthy aging (70). Out of the 105 mediators addressed, CD31, PDGF-AA and CD30 are the three more upregulated over Tet_off_ levels. We corroborate this finding at the mRNA level using a sensitive qPCR approach, with consistent results to previous gene microarray data, which also showed increased *TNFRSF8* (CD30) and *PECAM1* (CD31) mRNA levels on the same Jurkat Tat_101_ cells (38).

CD31 is a member of the immunoglobulin superfamily and influences leukocyte migration, angiogenesis, and integrin activation. *PECAM1*, is a member of the SenMAYO geneset, used to identify senescent cells across tissues (57). The expression of CD31 is high in naïve T-cells, especially in recent thymic emigrants, and decreases during T-cell activation (71). However, CD31 remains elevated during T-cell differentiation in HIV infection, which may result in exhausted T-cell functionality (72). We found *PECAM1* as an upregulated DEG in the database of an RNA-seq approach performed on a Jurkat Tet_on_ model of Tat expression (44), which suggests that *PECAM1* is upregulated early upon Tat expression and remains high upon chronic Tat exposure, as we see in our Jurkat Tet_off_ model. Importantly, elevated CD31 levels are found in the serum of PWH, which may contribute to blood-brain barrier permeability and neuro-AIDS (73).

CD30 is a TNF-receptor superfamily member, expressed in activated or in transformed cells, and very rarely on naïve T-cells (74). Importantly, CD4^+^ CD30^+^ cells are increased during HIV infection, both in c-ART treated or untreated individuals (75). *Hogan* et al. show that FACS-sorted CD4^+^ CD30^+^ from PWH express higher levels of HIV RNA than their CD30^-^ counterparts, even in ART-suppressed individuals. Further, increased levels of cell surface CD30 are detected in CD4^+^ T-cells from PWH with a viral load rebound upon an analytical treatment interruption (ATI). Critically, changes in CD30 expression are detected even before changes in plasma viral load occur (76). Older studies had already shown that crosslinking cell surface CD30 in the chronically HIV-infected cell line ACH2 triggered HIV expression without cell proliferation and that NF-κB signaling was required for this process (77). As HIV-Tat is critical for HIV transcription and increases NF-κB signaling, it is plausible that changes in CD30 in those studies are linked to HIV-TAT activity. Increased serum levels of sCD30 are a predictor of AIDS progression (78). Importantly, the stark increase in cell surface CD30 and CD31 co-expression in Tat_101_ cells compared to Tet_off_ and to Tat_72_, allows the discrimination of full-length Tat-expressing cells. This would allow therapeutic targeting strategies such as chimeric antigen receptor T-cells or antibody-based approaches (65, 79).

Additionally, we found an important increase in the secretion of platelet-derived growth factor AA subunit (PDGF-AA). This growth factor is important in angiogenesis and wound healing and is a SASP factor secreted by senescent skin cells (80). Previous studies show that soluble HIV-Tat causes an upregulation of the related PDGF-BB in endothelial cells, astrocytes, and pericytes, with an important role for NF-κB and ROS signaling pathways in this effect (81). In addition, increased mRNA levels of the PDGF/VEGF growth factor family members (*PDGFA, VEGFA-C*) are reported in transformed human mammary cells after HIV-Tat addition (82). Increased levels of PDGF during HIV infection contribute to the loss of blood-brain barrier integrity and neurological manifestations of HIV infection (81).

We took advantage of an RNA-seq performed in our Jurkat-Tat model (56) to address additional senescence-related transcripts and pathways that may be altered by Tat_101_ expression. The pathway analysis performed by *Rodríguez-Agustín* et al. unveils that one of the most up-regulated pathways in Tat_101_ expressing cells is the p53 pathway and one of the most downregulated is ribosome biogenesis. These pathways are both linked to cellular senescence onset (69, 83). Here, we looked up specific DEG genes in the Tat_101_ vs Tet_off_ comparison that are present in different senescence databases. We validated some of the targets found in our work, such as *CDKNA1* (p21), *PECAM1* (CD31), *PDGFA* (PDGF-AA), CCL1, and *TNFRSF8* (CD30). Importantly, *CDKNA1* (p21) is a critical senescence initiator under p53 regulation, underscoring the importance of this pathway for Tat-mediated effects on cellular senescence. We also find a striking (38 and 20-fold) increase in *CCND2* and *CCND1* genes and a 2-fold increase in *CCNE1*. These genes encode for cyclins D and E, whose expression is increased in senescent cells (84, 85) and are among the top 20 (out of 504) ranked cellular senescence genes screened in a literature-based gene resource (86). In this same study, senescence-related genes (504) are over-represented in processes related to HIV, and the top 4 enriched diseases of all senescence genes are all related to HIV transcription from LTR promoter and Tat activity (86). Our analysis also uncovers a transcriptional downmodulation of targets such as *IGFBP7, IGFBP2, HIST1H4C, RB1 or TIMP2*, which are usually upregulated in senescent cells at the protein level (43, 69). This suggests that HIV-Tat increases some, but not all the senescence biomarkers addressed. These markers could be altered by other HIV-derived proteins or by HIV infection progression.

Our work shows that Tat full-length protein is required for most of the effects on cellular senescence targets addressed. The 72aa, one exon form of Tat, can transactivate the LTR promoter in Jurkat cells, albeit with a reduced efficiency (38). Introducing a stop codon in position 72 in the Tat open reading frame of the HIV 89.6 infectious clone, results in viruses expressing a 72aa form of Tat, which show a significantly reduced replication rate than its wild-type counterpart (40). Here we show that Tat_72_ modestly increases p21 in the Jurkat cell line, but does not change BCL2, γH2AX or CD87 expression. In addition, Tat_72_ had a limited effect on SASP secretion. For instance, the secretion of CD30 and CD31 was upregulated 2.71 and 1.94-fold respectively, in Tat_101_ over Tet_off_, but only 1.42 and 1.44-fold respectively in Tat_72_ vs Tet_off_. Previous work determined that the ^86^ESKKKVE^92^ domain located in Tat_101_ second exon is important for the increased NF-κB signaling and protection from FasL-mediated apoptosis observed in Jurkat Tat_101_ (42, 49). These studies showed that Jurkat Tat_01_ cells markedly increased CD69 and BCL-2 expression, resistance to apoptosis, and overall NF-κB signaling over Tet_off_ cells. Additionally, increased ROS generation and mitochondrial dysfunction were observed in such cells (41), now considered hallmarks of cellular senescence (43). Jurkat Tat_72_ only modestly increased these parameters, which indicates that Tat’s second exon, is important for the Tat-mediated induction of cellular senescence and that NF-κB signaling pathway is involved in this process. Recent studies also show that the addition of an HIV-Tat protein containing only 66aa of the first exon to latently infected CD4^+^ T-cells resulted in a latency reversal effect without significantly altering the CD4^+^ T-cell transcriptome (87).

A limitation of our study is the transformed nature of the Jurkat cell line, which is homozygously deleted for *CDKN2A* (p16^INK4A^) and presents point mutations in other relevant genes such as *TP53 (*p53) (88). Despite this, plenty of studies showed senescence induction in transformed cells and in the Jurkat cell line (89, 90). We have also tested that extremely low doses of etoposide can induce the upregulation of senescence biomarkers in our Jurkat model. We do not observe changes in SA‐β‐gal staining in Tat_101_-expressing Jurkat cells. Cellular senescence is highly heterogeneous and may vary due to different inducers on different cell types (91). We delivered DNA plasmids encoding HIV-Tat to primary CD4^+^ T-cells which are the main target of HIV infection, to validate our findings in a relevant model. This strategy allowed the identification of live, Tat-expressing CD4^+^ T-cells by flow cytometry and the subsequent expression of senescent biomarkers in such cells. We thus confirmed that Tat expression upregulates p21, γH2AX and, importantly, p16 levels compared to Tat^-^ cells. This confirms our Jurkat cell data in a more physiological setting. Interestingly, previous work showed that p16 levels increased after exposing CD4^+^ T-cells to recombinant HIV-Tat for 24h (92). Authors suggest that this may be caused by the inhibition of telomerase activity (93) and reduced nuclear hTERT levels caused by HIV-Tat (92), further strengthening the idea that HIV-Tat induces cellular senescence in infected CD4^+^ T-cells.

Regarding other cell types, HIV-Tat has been shown to reduce phagocytosis in primary monocytes and macrophages, reducing its effectiveness against pathogens (94). Furthermore, HIV-Tat increases SA-βGAL, p21, IL6, IL8, and ROS generation in microglia (35, 59).Thus, we expect HIV-Tat to increase cellular senescence in other immune cells, like monocytes or macrophages. Interestingly, we observe that delivering Tat_72_ to CD4^+^ T-cells increases senescence biomarkers to a similar extent to Tat_101_. This could be explained by very high levels of HIV-Tat mRNA transcripts 24 hours after CD4^+^ T-cell electroporation (data not shown), presumably due to the strong CMV promoter in our DNA plasmids. These increased levels of Tat_72_ may be sufficient to increase senescence biomarkers. Our approach is based on resting T-cells, as a strong T-cell activation may modify senescence biomarker expression and difficult detection of Tat-mediated changes. As a consequence, the expression of some senescence biomarkers, such as CD87 is very low, and CD30 is nearly undetectable (75) in this model, so we could not fully assess changes in such markers. However, levels of secreted CD31 are increased in cell supernatants of Tat-transfected CD4^+^ cells, suggesting that Tat acts similarly to our Jurkat model. Both CD31 and CD26 levels are increased in serum from HIV-infected individuals (72). CD26 has been reported as a surface marker of senescent fibroblasts (95) and both CD31 and CD30 cleavage from cell surface leads to its presence in extracellular media. Importantly SASP expressing cells interacted with T-cells predominantly via the MHC-I, MIF and CD31 (PECAM1) pathways (57) underscoring the importance of these targets for senescent cells.

Recently, delivery of Tat RNA to CD4 T-cells by lipid nanoparticle constructs resulted in far superior efficiency and viability than traditional RNA transfection or soluble Tat addition (87, 96). We believe that this strategy deserves further consideration for future cellular senescence studies. An important point regarding the relevance of our findings is Tat mRNA in stable Jurkat cell lines is around 25-fold higher than in activated CD4^+^ T-cells. However, it should be taken into account that nearly all Jurkat cell lines express Tat, whereas approximately 1-10% of CD4 T-cells may be infected in the conditions tested. Accordingly, this data suggests that in a single CD4 lymphocyte actively replicating, HIV Tat expression may reach similar levels to Jurkat cells, which may lead to the induction of a senescence program in CD4 T cells. In summary, HIV-Tat increases senescence biomarkers in T-cells, possibly by increased DDR, p53, and NF-κB signaling. These effects and the increased SASP mediators released may have an important impact on chronic inflammation, blood-brain barrier permeability, neurological manifestations and other comorbidities seen in PWH, which may critically contribute to the premature aging phenotype of PWH. Importantly, the expression of Tat is largely unaffected by c-ART (97), and Tat is found in serum and cerebrospinal fluid of some ART-treated and virally suppressed individuals (45), which suggests that Tat may continue inducing senescence in those individuals, despite therapy. Thus, HIV-Tat is a relevant therapeutic target. Specific inhibitors of Tat exist (98, 99), which block the transactivation function of Tat. In addition, intradermal therapeutic vaccines using HIV-Tat as an immunogen have been quite successful at inducing HIV-Tat neutralizing antibody responses and reducing HIV proviral DNA in blood, even after 8 years of follow-up (100). It would be of great interest to evaluate senescence biomarkers in PWH with detectable levels of HIV-Tat in serum and those successfully responding to an HIV-Tat vaccine. All these data confirm HIV-Tat as a critical therapeutic target and an important driver of HIV pathogenesis and age-related pathologies in PWH.

## Data Availability

The data presented in the study are deposited in the GEO repository, accession number GSE282545.
