# Supplementary material for: HIV-Tat upregulates the expression of senescence biomarkers in CD4+ T-cells
Source: Front Immunol. 2025 Apr 24;16:1568762. doi: 10.3389/fimmu.2025.1568762 (PMC12058733; doi:10.3389/fimmu.2025.1568762)
Supplement: Supplementary file 1 [file DataSheet1.docx]

Supplementary Material

**
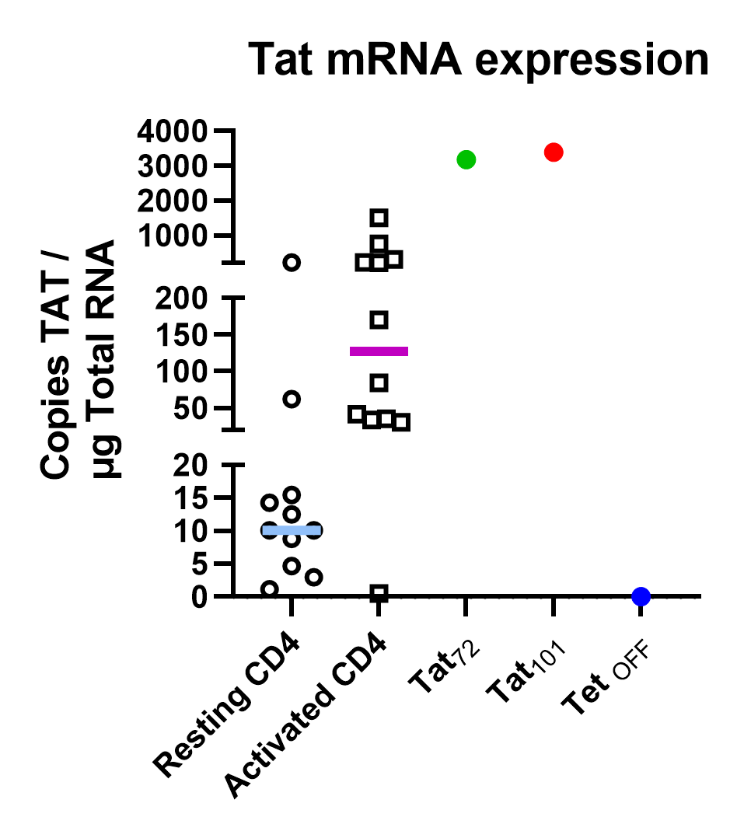
Supplementary Figure 1**

**Supplementary Fig 1: Comparison of Tat mRNA levels between PWH CD4^+^ T-cells and Jurkat stable cell lines.**

CD4^+^ T-cells were negatively isolated from cryopreserved PBMCs from PWH under ART treatment. Cells were rested or activated with Dynabeads™ Human T-Expander CD3/CD28 and IL-2 (100UI/mL) for 72h. RNA isolation was performed on CD4 or in Jurkat cell pellets (Rneasy® MiniKit (Qiagen). Retrotranscription and cDNA amplification was performed with the One-Step RT-ddPCR Advanced Kit for Probes (Bio-Rad). Tat mRNA was quantified in CD4^+^ T-cells using Tat/Rev primers and probe (FAM) (Yukl SA, 2018) with an annealing temperature of 54ºC. For Jurkat cells the ‘TAT72’ primers and probe (FAM) already described in the methods section were used (annealing of 60º). ddPCR was performed in a QX600 Droplet Digital PCR System (Bio-Rad). In all conditions, a housekeeping CD3 gene (HEX) was included to control for mRNA presence. Analysis was performed by adjusting ddPCR RNA raw data concentrations to RNA quantity and concentration in each well. Results are expressed as copies of Tat per μg of total RNA. Data represents individuals with detectable levels of Tat (11 out of 13 in Resting CD4 and 12 out of 13 in Activated CD4) Each symbol indicates a different individual (n=13) and lines indicate the median value of each group.

**
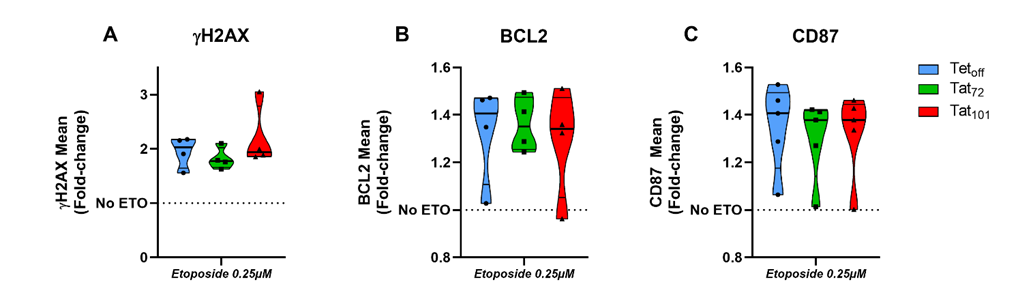
Supplementary Fig 2**

**Supplemental Fig 2. Low levels of Etoposide induce cell senescence in the different Jurkat cell lines.**

Jurkat Tet_off,_ Tat_72_ or Tat_101,_ were treated with Etoposide at 0.25 μM for 24 hours or left untreated (No ETO, dotted line). Flow cytometry was used to address cellular senescence biomarkers. The geometric mean of γH2AX **(A),** BCL2 **(B),** and CD87 **(C)** in the live cells gate (Live/Dead Negative) for the different conditions was measured. The fold change in senescence markers' expression in etoposide-treated cells compared to their untreated counterpart is shown. Floating Bars indicate minimum to maximum values with a line at the mean value. Symbols indicate individual experiments.

**Supplementary Figure 3**


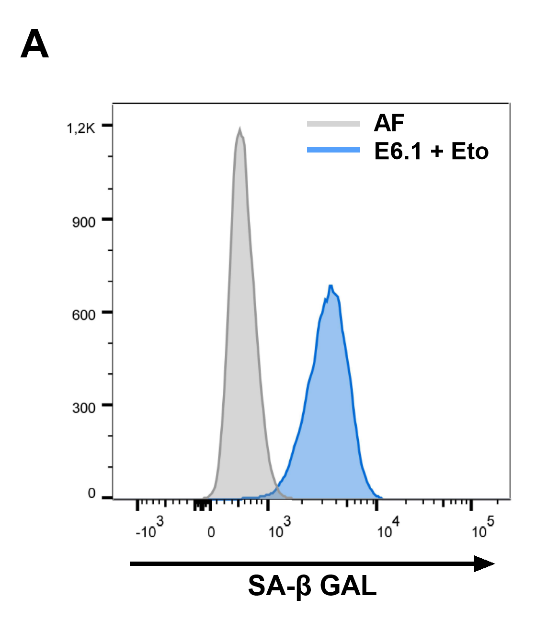


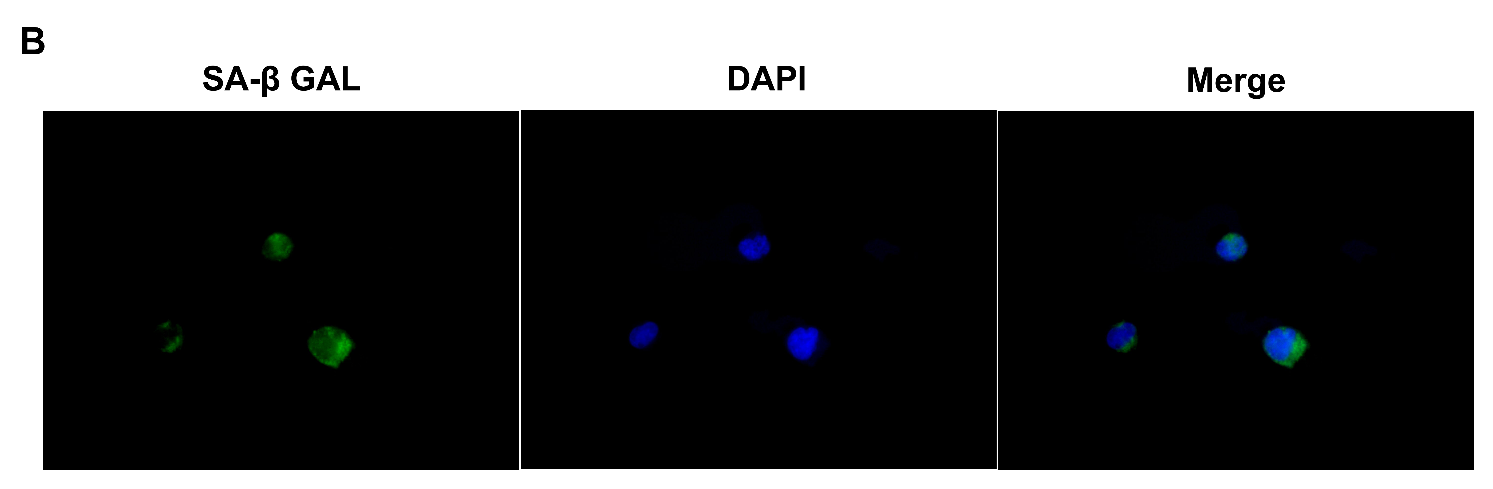


**Supplemental Figure 3**. Visualization of SA-βGal-stained Jurkat T cells. **(A)** Histogram overlays showing SA-βGal levels in Jurkat E6.1 cells treated with 0,25 uM Etoposide (E6.1+ETO) for 24H and cell Autofluorescence (AF). **(B)** Microscopy visualization of SPIDER SA-βGal fluorescence staining (in green) in the same Jurkat E6.1 cells shown in (A). Cells were laid in microscope slides and cover-slipped with mounting medium containing DAPI for nuclei visualization. Images were captured using a Zeiss Axiovert 200 inverted fluorescence microscope at 400X and analyzed with Image J software. Images show a mainly cytoplasmatic distribution of SPIDER SA-βGal staining. Representative results of n=3 independent experiments.

**Supplementary Figure 4**


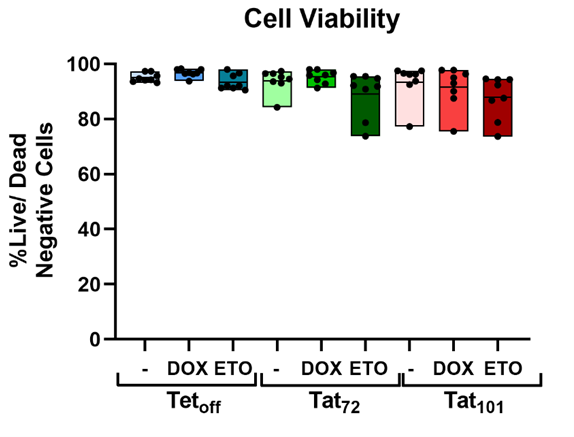


**Supplemental Fig 4. DOX or ETO treatments do not alter cell viability.**

Jurkat Tet_off,_ Tat_72_ or Tat_101,_ were treated with 1μg/ml Doxycycline (+DOX), or treated with Etoposide at 0.25 μM for 24hours (+ETO) or left untreated, and cell viability was addressed by Flow Cytometry (Live / Dead Negative staining). Floating Bars indicate minimum to maximum values with a line at the mean value. Symbols indicate individual experiments.

**Supplementary Figure 5**


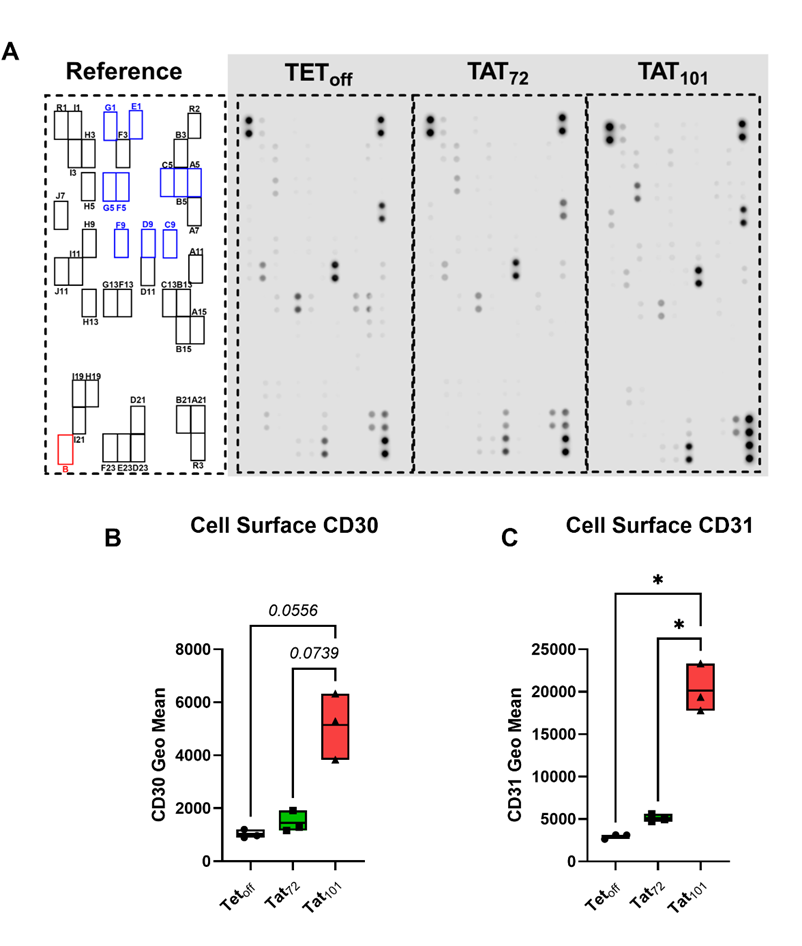


**Supplemental Fig 5. Visible mediators and their coordinates in a membrane-based cytokine array of Jurkat cell-supernatants.**

Jurkat Tet_off,_ Tat_72_ or Tat_101_ cell lines were grown for 72h and the cell-free-supernatant was addressed with a Proteome Profiler Human XL Cytokine Array Kit. Boxes with coordinates indicate the position of visible spots (in duplicate) after ECL incubation and image acquisition**.** Boxes labeled **R1-R3** indicate printed positive controls and coordinates labeled **B** indicate the position used to determine background levels. The geometric mean of cell surface CD30 **(B)** and CD31 **(C)** in the Live Cells Gate (Live/Dead^-^) was addressed by Flow Cytometry after 24 hours of cell culture. Symbols indicate individual experiments (n=3), with Floating Bars showing minimum to maximum values with a line at the mean. Statistical analysis was performed by one-way ANOVA with Tukey’s multiple comparisons test. *p ≤ 0.05.

**Supplementary Figure 6**

**
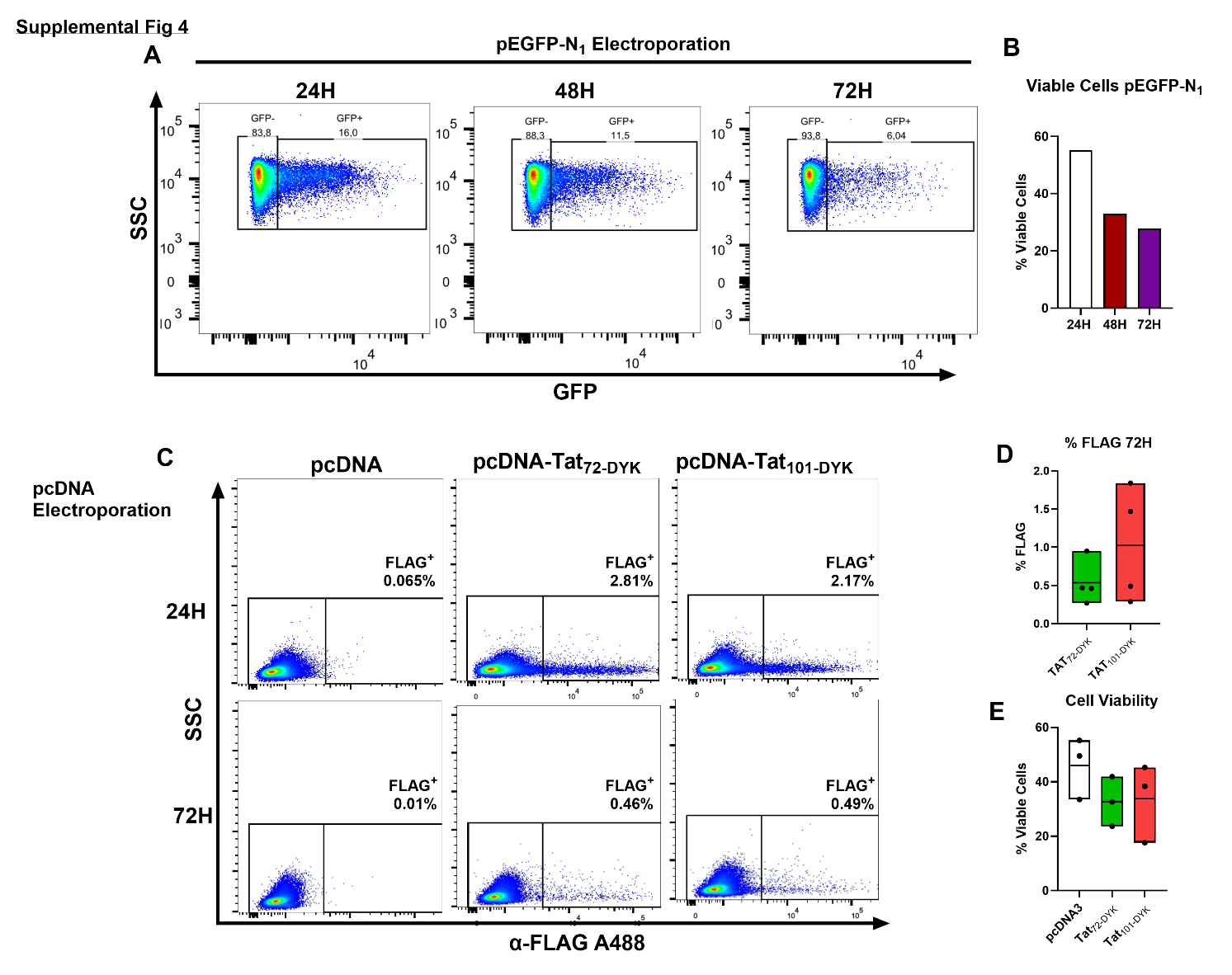
**

**Supplemental Fig 6. Primary, resting CD4^+^ T-cell electroporation efficiency.**

A total of 2 × 10⁶ CD4^+^ T cells were electroporated with 7.5 μg of DNA plasmids using a 100 μl NEON NXT tip and cultured in antibiotic-free R10 media for 24, 48, or 72 hours. Representative dot plots showing the % of GFP^+^ cells after pEGFP-N1 electroporation (indicative of transfection efficiency) in the live cells gate (Live / Dead^-^) are shown in **(A)**. The percentage of viable cells is shown in **(B).** pcDNA3 or pcDNA3-Tat-FLAG constructs were electroporated in resting CD4^+^ T-cells and FLAG levels addressed by Flow Cytometry. Dot Plots from a representative experiment showing anti-FLAG staining 24 or 72h after electroporation in shown in **(C).** Bars indicating the percentage of Tat-FLAG^+^ cells in the Live cells gate 72h after electroporation of Tat_72-DYK_ or Tat_101-DYK in_ four different experiments are shown. The percentage of viable cells for these electroporations (Live/Dead negative gate) was addressed by Flow Cytometry and shown in **(E).** Symbols indicate individual experiments, with floating Bars showing minimum to maximum values with a line at the mean.
